# A theoretical and experimental proteome map of *Pseudomonas aeruginosa* PAO1

**DOI:** 10.1002/mbo3.21

**Published:** 2012-06

**Authors:** Elke Lecoutere, Peter Verleyen, Steven Haenen, Katrien Vandersteegen, Jean-Paul Noben, Johan Robben, Liliane Schoofs, Pieter-Jan Ceyssens, Guido Volckaert, Rob Lavigne

**Affiliations:** 1 Faculty of Bioscience Engineering,, Division of Gene Technology, Department of Biosystems, Katholieke Universiteit LeuvenBelgium; 2 Research Group of Functional Genomics and Proteomics, Katholieke Universiteit LeuvenBelgium; 3 Biomedical Research Institute, UHasseltBelgium; 4 Present address: Department of Biochemistry, Molecular and Structural Biology, Katholieke Universiteit LeuvenBelgium

**Keywords:** Mass spectrometry (MS), proteomics, two-dimensional gel electrophoresis (2-DE)

## Abstract

A total proteome map of the *Pseudomonas aeruginosa* PAO1 proteome is presented, generated by a combination of two-dimensional gel electrophoresis and protein identification by mass spectrometry. In total, 1128 spots were visualized, and 181 protein spots were characterized, corresponding to 159 different protein entries. In particular, protein chaperones and enzymes important in energy conversion and amino acid biosynthesis were identified. Spot analysis always resulted in the identification of a single protein, suggesting sufficient spot resolution, although the same protein may be detected in two or more neighboring spots, possibly indicating posttranslational modifications. Comparison to the theoretical proteome revealed an underrepresentation of membrane proteins, though the identified proteins cover all predicted subcellular localizations and all functional classes. These data provide a basis for subsequent comparative studies of the biology and metabolism of *P. aeruginosa*, aimed at unraveling global regulatory networks.

## Introduction

The pseudomonads comprise a group of Gram-negative bacteria with a high metabolic versatility allowing them to adapt to a broad range of environmental niches. *Pseudomonas aeruginosa* is an opportunistic pathogen responsible for severe life-threatening infections in immunocompromised patients. For example, in individuals with cystic fibrosis, chronic colonization of the lung mucosa by *P. aeruginosa* is a major cause of death (Govan and Deretic 1996; Lyczak et al. 2002; Ratjen and Doring 2003).

*Pseudomonas aeruginosa* possesses a strong inherent antibiotic resistance, partly due to extensive efflux systems and a highly impermeable membrane (Ahmad 2002). In addition, an increasing number of *P. aeruginosa* strains have developed an alarming level of acquired antibiotic resistance, caused by their large and adaptable genome, which, in combination with the development of impermeable biofilms, creates an even greater challenge in the battle against *P. aeruginosa* infections (Hancock and Speert 2000; Singh et al. 2000; Stewart and Costerton 2001; Drenkard 2003). Given its importance as a human pathogen, *P. aeruginosa* represents a useful model organism. Moreover, the availability of the completed 6.3-Mbp genome of *P. aeruginosa* PAO1 (Stover et al. 2000), revealing 5570 annotated Open Reading Frames (ORFs) (PseudoCAP) (Winsor et al. 2005), offers the opportunity to perform extensive proteome analyses.

In the past, studies have focused on disrupting biofilms and identifying new intracellular targets to develop novel classes of antibiotics (Stewart and Costerton 2001). Proteomic studies provide more insight into gene function and will play a vital role in unraveling the basic biology of microorganisms. Several recent *P. aeruginosa* studies using two-dimensional gel electrophoresis (2-DE) aimed at both exploring the adaptation of the organism under nutrient and oxygen limitation (Hummerjohann et al. 1998; Quadroni et al. 1999; Guina et al. 2003; Heim et al. 2003; Wu et al. 2005b; Siqueira Reis et al. 2010) and at understanding of virulence (Hanna et al. 2000; Termine and Michel 2009), biofilm formation (Yoon et al. 2002; Southey-Pillig et al. 2005; Nigaud et al. 2010), and quorum-sensing signals (Arevalo-Ferro et al. 2003).

Here, the cytoplasmic 2-D reference map of the *P. aeruginosa* PAO1 proteome is presented, complementing the previously mapped *P. aeruginosa* membrane proteome (Nouwens et al. 2000) and periplasmic proteome (Imperi et al. 2009). 2-DE provides the reproducibility required for creating a reliable reference map, in combination with MALDI-TOF, MALDI-TOF/TOF, and ESI-MS/MS for protein identification. The experimental and theoretical proteome were compared using the data generated from the 181 identified protein spots. The proteome map presented here may serve as a reference for future studies, allowing comparative analyses for a variety of *Pseudomonas* strains under diverse conditions.

## Materials and Methods

### Bacterial strain and protein extraction

*Pseudomonas aeruginosa* strain PAO1 (Stover et al. 2000) cells were grown aerobically under vigorous agitation at 37°C in LB broth (10 g/L tryptone, 5 g/L yeast extract, 10 g/L NaCl) to exponential phase (OD_600 nm_ ≅ 0.6). For protein extraction, 20 mL of the bacterial culture was pelleted (3000 *g*, 4°C, 30 min), washed three times with Tris-HCl (25 mM, pH 7.5), and resuspended in 2 mL lysis buffer (7 M urea, 2 M thiourea, 4% CHAPS, 2% IPG buffer, 40 mM DTT) containing protease inhibitors (Protease Inhibitor Mix, GE Healthcare, Sweden), Na_2_EDTA (5 mM), and DNaseI (0.1 mg/mL). Subsequently, cell disruption was improved by sonication on ice, cell debris was pelleted (3000 *g*, 4°C, 1 h), and the supernatant was collected for storage at –80°C. Total protein concentration of the samples was evaluated using the 2-DE Quant Kit (GE Healthcare). Four samples of independent cultures were made.

### 2-DE and image analysis

All 2-DE separations and image analyses were carried out using GE Healthcare devices and reagents. Iso Electric Focusing was performed using IPG strips (24-cm Immobiline DryStrips with linear pH gradient range 3–10 or 4–7). The strips were rehydrated overnight in a denaturating reswelling solution (7 M urea, 2 M thiourea, 2% w/v CHAPS, DeStreak Reagent, 0.5% IPG buffer, and a trace of bromophenol blue). The samples were applied by anodic cup-loading, and IEF was performed in the Ettan IPGphorII according to Görg et al. (2000). Following IEF, proteins were reduced and alkylated as described by Bae et al. (2003) using equilibration buffer I and II (6 M urea, 30% w/v glycerol, 2% w/v SDS in 50 mM Tris-HCl, pH 8.8) containing 1% DTT and 4% iodoacetamide (IAA), respectively. Subsequently, the second dimension (SDS-PAGE) was run in 1-mm thick vertical gels (15% polyacrylamide) using the Ettan DALT*six* (GE HealthCare, UK). Protein spots were visualized by colloidal CBB G-250 staining (Neuhoff et al. 1988) or MS compatible silver nitrate staining (Shevchenko et al. 1996). Image acquisition was performed using a calibrated flatbed ImageScanner, combined with LabScan software. 2-DE maps were analyzed and spot data generated using ImageMaster 2D Platinum software. For each biological sample, six replicate gels were made.

### In-gel protein digestion

Protein digestion was performed as detailed by Shevchenko et al. (1996). In short, Coomassie blue spots were excised from the gels and destained. The proteins were reduced and alkylated, whereafter the gel slices were sequentially hydrated and dried. Trypsin (Promega, Madison, WI) was added, followed by overnight digestion. Finally, peptides were extracted from the gel by sonication.

### Mass spectrometry

Prior to mass spectrometric analysis, peptide samples were dried in a vacuum centrifuge and desalted using ZipTip C_18_ pipette tips (Millipore, Bedford, MA). MALDI-TOF analyses were performed on a Reflex IV (Bruker Daltonik GmbH, Bremen, Germany) operating in reflectron mode. The matrix, consisting of saturated α-cyano-4-hydroxycinnamic acid in aceton, was cocrystallized with the peptide sample by the dried droplet technique. ESI-MS/MS was performed on an LCQ Classic (ThermoFinnigan, San Jose) equipped with a nano-LC column switching system as described previously (Dumont et al. 2004).

### Protein identification

Proteins were identified by searching the NCBI database using Sequest (ThermoFinnigan) and Mascot (Matrix Science, MA). One missed cleavage was allowed and a mass tolerance of 0.3 Da was used. Possible modifications such as carbamidomethylation of cysteine and oxidation of methionine were included. For unambiguous peptide-mass fingerprint identification, more than five peptides must be matched and the sequence coverage must be greater than 15%. Agreement between theoretical and experimental p*I* and *M_r_* was also taken into account.

### In silico analysis

All calculations were based on the 5570 annotated protein sequences included in the database of *P. aeruginosa* PAO1 (PseudoCAP) (Winsor et al. 2005). This database also provided information about predicted cellular localization and cluster of orthologous groups (COG) functional categories. The physical parameters of the proteins were computed with the ProtParam Tool at the ExPASy server (Gasteiger et al. 2005), calculating the theoretical p*I* as described by Bjellqvist et al. (1993) and the grand average of hydrophobicity (GRAVY) according to Kyte and Doolittle (1982). The codon adaptation index (CAI) of identified proteins was measured by the CAI calculator (Wu et al. 2005a) using the equation of Sharp and Li (1987) and a codon usage template of highly expressed genes (Grocock and Sharp 2002). Signal peptides were predicted using SignalP 3.0 (Brendtsen et al. 2004). Parameter statistics were performed by online QuickCalcs tools.

## Results and Discussion

### Theoretical *P. aeruginosa* PAO1 proteome

The relatively large genome of *P. aeruginosa* (6.3 Mbp) contributes to its high versatility and environmental adaptability. With 5570 annotated genes, *P. aeruginosa* PAO1 is capable of expressing a proteome comparable in size and complexity to lower eukaryotes such as *Saccharomyces cerevisiae* (Stover et al. 2000).

Because physical parameters can be predicted from protein sequences using web-based tools, exploring the properties of the theoretical *P. aeruginosa* proteome allows to choose appropriate conditions for 2-DE. Although these properties may be altered by posttranslational modification for a minority of the proteins, typically, isoelectric point (p*I*) and relative molecular mass (*M_r_*) can be accurately calculated.

### Relative molecular mass (*M_r_*)

**Figure 1:**
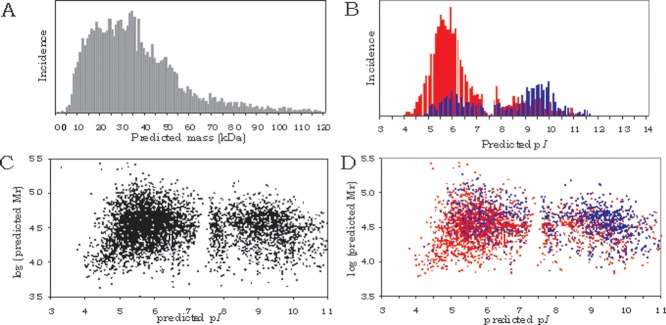
Predicted parameters and virtual 2D-gel of the *Pseudomonas aeruginosa* proteome. (A) The predicted mass distribution is unimodal. (B) The predicted charge distribution is bimodal with a minor third peak. The p*I* of cytoplasmic proteins (red) is typically lower than the p*I* of membrane proteins. (C) The pattern on a virtual two-dimensional gel electrophoresis (2-DE) gel has a butterfly appearance. To obtain a general overview, IPG strips with pH 3–10 will be used. (D) In the virtual 2D-gel, the shift of cytoplasmic proteins (red) toward lower p*I*, and membrane proteins (blue) toward higher p*I* is observed again.

The 5570 annotated *P. aeruginosa* proteins show a unimodal mass distribution with the majority of protein masses between 10 and 50 kDa, with a long tail up to 120 kDa (Fig. 1A). This proteome consists of only 239 small (<10 kDa) and 126 large (>100 kDa) proteins, while the remainder 93% has an *M_r_* suitable for regular 2-DE. Hence, no adaptation of standard 2-DE methods was needed.

### Isoelectric point (p*I*)

The predicted isoelectric points for the 5570 *P. aeruginosa* proteins were calculated and showed a bimodal charge distribution with peaks around p*I* 5.5 and 9.5. An additional minor peak is visible around p*I* 7.8, while almost no proteins have a p*I* near 7.5 (Fig. 1B). The majority of *P. aeruginosa* proteins (64%) have p*I*-values between 4 and 7, while only 5% fall outside the range of commercial IEF strips (3–11). Taking into account the predicted protein sublocalization, a shift toward the acidic region for cytoplasmic proteins (mean p*I* = 6.36) and toward the alkaline region for predicted inner membrane proteins (mean p*I* = 8.11) is observed (Fig. 1B). The shift is universal among all three domains of life (Schwartz et al. 2001). The significantly higher (*P* < 0.0001) p*I*-value of membrane proteins is consistent with the fact that most biomembranes have negatively charged surfaces (Schwartz et al. 2001).

### Theoretical 2-DE gel

Virtual 2-DE gels are generated by plotting the theoretical *M_r_* against the theoretical p*I*. A map was made using a linear scale on the *x*-axis to imitate protein mobility during isoelectric focusing and a logarithmic scale on the *y*-axis to represent protein migration during SDS-PAGE. The p*I* range was set from 3 to 11 and the *M_r_* range from 3 to 300 kDa (Fig. 1C). The theoretical proteome plot reveals a “butterfly-distribution,” the left wing consisting of acidic proteins, the right wing of alkaline proteins. The body part represents the minor peak near pH 8. This pattern was previously reported for *Escherichia coli* (Link et al. 1997b; VanBogelen et al. 1997) as well as for other bacteria (Link et al. 1997a; Urquhart et al. 1997; Drews et al. 2004) and appears to be similar for proteomes in all three domains of life (Archaea, Eubacteria, and Eukarya) (Knight et al. 2004; Weiller et al. 2004). The near absence of proteins with cytoplasmic pH (between 7.2 and 7.4) (Urquhart et al. 1998) may be caused by avoidance of the intracellular pH, at which proteins are difficult to maintain in solution. Additionally, Schwartz et al. (2001) state that the p*I* bimodality may be the result of the need for different p*I*-values depending on subcellular localization, since membrane proteins have a significantly higher p*I*-value than cytoplasmic proteins. This hypothesis is supported by the fact that eukaryotes show trimodal p*I* distribution, with the third peak mainly consisting of nuclear proteins.

Considering subcellular localization of *P. aeruginosa* proteins, a shift in the virtual 2-DE gel toward the left and right side, for cytoplasmic and inner membrane proteins, respectively, is again observed (Fig. 1D). The membrane proteome of *P. aeruginosa* was mapped previously by Nouwens et al. (2000); this study mainly focuses on cytoplasmic proteins. The resolving power is enhanced by focusing on the p*I* range 4–7, within which the p*I* of the major part of cytoplasmic proteins falls (77.5%).

### 2-DE map of the *P. aeruginosa* proteome

**Figure 2:**
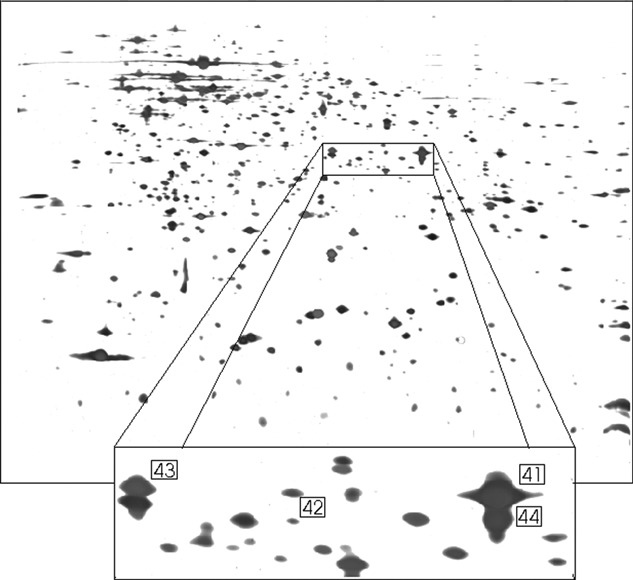
Two-dimensional gel electrophoresis (2-DE) reference map of the *Pseudomonas aeruginosa* proteome. This gel was silver stained. The box depicts an example of a protein appearing in mutiple spots: SucD was found in spots 41–44, with an experimental p*I* range 5.42–5.72. Other spot numbers are indicated in the Figure S1.

Optimal results for protein extraction were obtained using protease inhibitors, EDTA, and DNaseI. A total of 300–400 μg of proteins extracted from *P. aeruginosa*, exponentially growing on rich medium, were applied by anodic cup loading. To obtain a general overview, initial protein separations were performed on IPG strips with a pH range 3–10. As predicted, most visible protein spots were concentrated in the acidic region of the gel (95%). For higher resolution of cytoplasmic proteins, a switch to strips with a pH range of 4–7 was made. The estimated number of 2-DE detectable proteins with a p*I* between 4 and 7, an *M_r_* between 10 and 100 kDa, and low hydrophobicity (GRAVY < 0.400) is 3319. On the silver-stained gels, 1128 spots were detected using ImageMaster software (Fig. 2), accounting for approximately 33% of the theoretically detectable proteome. Under the used growth conditions, a total proteome expression is not expected. When making a general comparison with a similar expression analysis in *E. coli* (Richmond et al. 1999), the relative number of expressed housekeeping genes compared to the total number of gene products is consistent.

### Protein identification

In the reference gel with pH range 4–7, a random subset of spots distributed over the two-dimensional map were selected. One hundred and eighty one spots were unambigously identified by MS, originating from 159 different protein species (Table 1; Fig. S1). Spot analysis always resulted in the identification of a single protein, although the same protein may be detected in two or more neighboring spots (as discussed below).

**Table 1:** List of proteins identified from *Pseudomonas aeruginosa* PAO1

SpoNo.†	PA no.‡	Gene	Protein description	COG¶	Loc.§	*Mr* (kDa)	*Mr*†† (kDa)	p*I*	p*I*††	CAI	GRAVY
1	PA0139	*ahpC*	Alkyl hydroperoxide reductase	O	C	20.5	25	5.89	5.96	0.896	−0.182
2*	PA0143	*nuh*	Ribonucleoside hydrolase	F	P	37.5	37	7.03	6.24	0.687	0.029
3	PA0165		Hypothetical protein	M	OM	31.4	30	5.08	4.95	0.706	−0.443
4*	PA0291	*oprE*	Outer membrane porin	/	OM	49.7	43	8.67	6.22	0.796	−0.436
5*	PA0292	*aguA*	Agmatine deiminase	E	C	41.2	47	4.84	4.79	0.697	−0.495
6	PA0301	*spuE*	Polyamine transport	E	P	40.1	38	5.51	5.24	0.683	−0.193
7	PA0330	*rpiA*	Ribose 5-phosphate isomerase	G	C	23.7	26	5.38	5.38	0.702	0.137
8	PA0381	*thiG*	Thiamine biosynthesis protein	H	C	28.2	30	5.01	4.92	0.667	0.182
9	PA0409	*pilH*	Twitching motility protein	TK	C	13.3	10	5.35	5.22	0.697	−0.260
10*	PA0423	*pasP*	Secreted factor	S	U	20.8	21	6.09	5.49	0.807	−0.380
11	PA0446		Conserved hypothetical protein	C	C	43.8	44	5.36	5.32	0.651	−0.125
12	PA0546	*metK*	Methionine adenosyltransferase	H	C	42.7	45	5.26	5.19	0.815	−0.103
**13**	PA0552	*pgk*	Phophoglycerate kinase	G	C	40.4	40	5.28	5.13	0.727	0.206
**14**	PA0552	*pgk*	Phophoglycerate kinase	G	C	40.4	40	5.28	5.23	0.727	0.206
15	PA0555	*fda*	Fructose bisphosphate aldolase	G	C	38.6	41	5.34	5.39	0.804	−0.201
16	PA0607	*rpe*	Ribulose phosphate epimerase	G	C	24.1	23	5.16	5.08	0.745	0.208
17	PA0655		Hypothetical protein	H	C	23.6	26	5.39	5.55	0.697	−0.266
18	PA0664		hypothetical protein	M	C	14.8	12	5.54	5.53	0.512	−0.113
19	PA0668	*tyrZ*	tyrosyl tRNA synthetase	J	C	44.1	45	5.61	5.56	0.510	−0.192
20*	PA0766	*mucD*	Serine protease	O	P	50.3	50	7.04	6.11	0.646	−0.057
21	PA0796	*prpB*	Carboxyphophonoenolpyruvate phosphonomutase	G	C	32.1	34	5.33	5.27	0.765	0.032
22	PA0871	*phhB*	Pterin alpha carbinolamine dehydratase	H	C	13.3	12	5.94	5.96	0.607	−0.336
23*	PA0888	*aotJ*	Arginine/ornithine-binding protein	ET	P	28.0	28	6.43	5.31	0.715	−0.203
24	PA0895	*aruC*	N succinylglutamate semialdehyde dehydrogenase	E	C	43.7	42	5.63	5.61	0.706	−0.087
25	PA0932	*cysM*	Cysteine synthase B	E	U	32.4	31	5.54	5.51	0.637	−0.268
26	PA0945	*purM*	Phosphoribosylaminoimidazole synthetase	F	C	37.1	40	4.78	4.72	0.753	0.120
27	PA0956	*proS*	Prolyl tRNA synthetase	J	C	63.1	67	5.28	5.22	0.693	−0.194
28	PA0958	*oprD*	membrane porin	/	OM	48.4	47	4.96	4.71	0.645	−0.466
29	PA0962		DNA-binding stress protein	P	C	17.5	15	4.96	4.93	0.604	−0.123
30	PA0976		Conserved hypothetical protein	R	U	23.9	28	4.99	4.90	0.477	−0.001
31	PA0997	*pqsB*	Betaketoacyl carrier synthase	I	C	30.5	31	4.85	4.77	0.537	−0.050
32*	PA0999	*pqsD*	Oxoacylacyl carrier synthase	I	U	36.4	51	5.34	5.03	0.542	0.072
33	PA1010	*dapA*	Dihydrodipicolinate synthase	EM	C	31.4	32	6.00	5.99	0.705	0.043
34	PA1013	*purC*	Phosphoribosylaminoimidazole succinocarboxamide synthase	F	U	26.8	27	5.31	5.19	0.729	−0.306
35*	PA1074	*braC*	Branched chain amino acid transport protein	E	P	39.8	43	5.60	5.09	0.756	−0.155
36*	PA1084	*flgI*	Flagellar P-ring protein	N	P	38.2	39	6.92	5.99	0.694	0.129
37	PA1092	*fliC*	Flagellin type B	N	P	49.2	54	5.40	4.91	0.731	−0.077
38*	PA1288		Outer membrane protein	I	OM	45.6	40	5.73	5.36	0.738	−0.263
39*	PA1493	*cysP*	Sulfate-binding protein of ABC transporter	P	P	36.5	37	7.76	6.18	0.763	−0.332
40	PA1588	*sucC*	Succinyl CoA synthetase beta	C	C	41.5	41	5.83	5.82	0.814	−0.065
**41**	PA1589	*sucD*	Succinyl CoA synthetase alpha	C	U	30.3	32	5.79	5.72	0.854	0.192
**42**	PA1589	*sucD*	Succinyl CoA synthetase alpha	C	U	30.3	32	5.79	5.55	0.854	0.192
**43**	PA1589	*sucD*	Succinyl CoA synthetase alpha	C	U	30.3	33	5.79	5.42	0.854	0.192
**44**	PA1589	*sucD*	Succinyl CoA synthetase alpha	C	U	30.3	31	5.79	5.72	0.854	0.192
45	PA1597		Hypothetical protein	Q	U	25.8	30	5.66	5.69	0.583	−0.109
46	PA1609	*fabB*	Betaketoacyl ACP synthase	IQ	C	42.8	44	5.39	5.41	0.735	−0.087
47	PA1657		Conserved hypothetical protein	S	U	18.2	19	4.82	4.71	0.619	−0.330
48	PA1677		Conserved hypothetical protein	Q	C	21.0	21	6.05	5.97	0.545	0.005
49	PA1772		Methyltransferase	H	U	17.4	16	4.83	4.65	0.683	−0.011
50	PA1777	*oprF*	Major porin	M	C	37.6	40	5.02	4.78	0.831	−0.084
51*	PA1787	*acnB*	Aconitate hydratase	C	U	93.6	85	5.22	5.18	0.805	−0.104
52	PA1793	*ppiB*	Peptidyl prolyl cis-trans isomerase	O	C	18.1	16	5.79	5.83	0.816	−0.385
53	PA1796	*folD*	Methylene tetrahydrofolate dehydrogenase	H	C	30.5	31	5.58	5.65	0.689	0.053
54*	PA1800	*tig*	Trigger factor	O	C	48.6	54	4.83	4.76	0.699	−0.395
55	PA1837		Hypothetical protein	S	C	18.8	19	4.88	4.88	0.69	−0.378
56	PA2001	*atoB*	Acetyl CoA acetyltransferase	I	C	40.4	40	6.03	6.02	0.716	0.121
57	PA2064	*pcoB*	Copper resistance protein	P	U	35.3	36	4.85	4.70	0.608	−0.424
58	PA2081	*kynB*	Kynurenine formamidase	R	C	23.2	25	5.25	5.17	0.554	−0.060
59	PA2119		Alcohol dehydrogenase	ER	C	38.6	42	5.43	5.43	0.527	0.099
60	PA2505	*opdT*	Tyrosine porin	/	OM	49.8	51	4.91	4.68	0.678	−0.518
61	PA2532	*tpx*	Thiol peroxidase	O	U	17.2	17	5.16	5.13	0.770	0.207
62	PA2575		Hypothetical protein	R	U	22.2	22	5.96	5.96	0.690	−0.157
63	PA2614	*lolA*	Periplasmic chaperone	M	P	23.1	22	5.75	4.98	0.663	−0.154
**64**	PA2623	*icd*	Isocitrate dehydrogenase	C	C	45.6	45	5.10	5.00	0.736	−0.116
**65**	PA2623	*icd*	Isocitrate dehydrogenase	C	C	45.6	44	5.10	5.06	0.736	−0.116
**66**	PA2623	*icd*	Isocitrate dehydrogenase	C	C	45.6	47	5.10	4.93	0.736	−0.116
**67**	PA2623	*icd*	Isocitrate dehydrogenase	C	C	45.6	46	5.10	5.02	0.736	−0.116
68	PA2638	*nuoB*	NADH dehydrogenase	C	U	25.4	27	5.31	5.19	0.619	−0.238
69	PA2760		Outer membrane protein	/	OM	46.9	44	5.54	5.10	0.735	−0.467
70*	PA2800		Conserved hypothetical protein	M	U	26.1	25	5.41	4.90	0.691	−0.226
71	PA2806		Conserved hypothetical protein	S	C	30.8	30	5.48	5.45	0.569	−0.239
72*	PA2851	*efp*	Translation elongation factor P	J	C	21.0	27	4.82	4.85	0.702	−0.283
73	PA2951	*etfA*	Electron transfer flavoprotein	C	U	31.4	34	4.98	4.96	0.843	0.343
74	PA2965	*fabF1*	Betaketoacylacyl carrier synthase	IQ	C	43.5	45	5.62	5.64	0.654	−0.034
75	PA2967	*fabG*	Oxoacylacyl carrier reductase	IQR	C	25.6	24	6.16	6.09	0.604	0.176
76	PA2968	*fabD*	Malonyl CoA acyl carrier transacylase	I	U	32.4	32	5.05	5.00	0.595	0.253
77	PA3148	*wbpI*	UDP-N-acetylglucosamine 2-epimerase	M	C	38.9	38	5.64	5.63	0.350	0.056
78*	PA3162	*rpsA*	30S ribosomal protein S1	J	C	61.7	67	4.83	4.74	0.631	−0.343
79	PA3165	*hisC2*	Histidinol-phosphate aminotransferase	E	C	39.5	41	5.05	5.01	0.553	0.058
80	PA3167	*serC*	3-phosphoserine aminotransferase	HE	C	39.9	43	4.96	4.92	0.598	−0.238
81	PA3173		Short-chain dehydrogenase	IQR	C	26.3	29	5.09	5.12	0.692	−0.031
82	PA3190		Sugar-binding ABC transporter	G	P	45.1	41	5.68	5.28	0.694	−0.143
83	PA3244	*minD*	Cell division inhibitor	D	CM	29.6	30	5.58	5.57	0.626	−0.114
84	PA3302		Conserved hypothetical protein	I	U	16.9	19	5.24	5.21	0.642	−0.057
85	PA3309		Universal stress protein	T	U	16.5	18	5.50	5.42	0.729	−0.125
86	PA3480		Deoxycytidine triphophate deaminase	F	C	21.2	23	5.95	6.00	0.722	−0.252
87	PA3481		Conserved hypothetical protein	D	U	38.9	40	5.27	5.17	0.648	0.090
**88**	PA3529		Peroxidase	O	C	21.8	23	5.37	5.33	0.809	−0.077
**89**	PA3529		Peroxidase	O	C	21.8	23	5.37	5.18	0.809	−0.077
90	PA3610	*potD*	Polyamine transport	E	P	39.3	36	5.57	5.26	0.664	−0.225
91	PA3635	*eno*	Enolase	G	C	45.2	48	5.05	5.04	0.802	−0.142
92	PA3639	*accA*	Acetyl CoA carboxylase	I	CM	34.9	38	5.34	5.50	0.660	−0.269
93	PA3646	*lpdX*	UDP-hydroxyauroyl glucosamine acetyltransferase	M	U	36.2	40	5.84	5.83	0.571	0.187
94*	PA3648		Outer membrane protein	M	OM	88.3	82	5.05	4.89	0.753	−0.368
95	PA3653	*frr*	Ribosome recycling factor	J	C	20.5	22	5.85	5.73	0.704	−0.464
**96**	PA3655	*tsf*	Elongation factor Ts	J	C	30.6	33	5.22	5.13	0.807	−0.020
**97**	PA3655	*tsf*	Elongation factor Ts	J	C	30.6	34	5.22	5.07	0.807	−0.020
**98**	PA3655	*tsf*	Elongation factor Ts	J	C	30.6	35	5.22	4.87	0.807	−0.020
99	PA3666	*dapD*	Tetrahydrodipicolinate succinylase	E	U	35.8	38	5.74	5.69	0.751	0.208
100	PA3770	*guaB*	Inosine monophosphate dehydrogenase	F	U	51.7	52	6.24	6.17	0.781	0.027
101	PA3801		Conserved hypothetical protein	S	U	23.1	24	5.00	4.87	0.658	−0.408
102	PA3807	*ndk*	Nucleoside diphosphate kinase	F	C	15.6	13	5.48	5.45	0.788	−0.089
103	PA4007	*proA*	Gamma glutamyl phosphate reductase	E	C	45.0	45	5.33	5.33	0.693	−0.015
**104**	PA4031	*ppa*	Inorganic pyrophosphatase	C	C	19.4	23	5.04	4.87	0.730	−0.142
**105**	PA4031	*ppa*	Inorganic pyrophosphatase	C	C	19.4	23	5.04	4.99	0.730	−0.142
106	PA4053	*ribE*	Dimethyl ribityllumazine synthase	H	CM	16.4	13	5.69	5.68	0.825	0.449
107	PA4061		Thioredoxin	O	C	31.9	32	4.63	4.63	0.716	−0.101
**108**	PA4067	*oprG*	Outer membrane protein	M	OM	25.2	25	4.85	4.64	0.770	−0.105
109*	PA4067	*oprG*	Outer membrane protein	M	OM	25.2	18	4.85	4.50	0.770	−0.105
110	PA4232	*ssb*	ssDNA-binding protein	L	U	18.6	19	5.46	5.44	0.672	−1.028
111*	PA4238	*rpoA*	DNA-directed RNA polymerase	K	C	36.6	44	4.88	4.93	0.633	−0.249
112*	PA4238	*rpoA*	DNA-directed RNA polymerase	K	C	36.6	44	4.88	5.15	0.633	−0.249
113	PA4265	*tufA*	Elongation factor Tu	J	C	43.4	44	5.23	5.06	0.806	−0.152
114	PA4266	*fusA1*	Elongation factor G	J	C	77.8	78	5.06	4.61	0.721	−0.307
115*	PA4271	*rplD*	50S ribosomal protein L7/L12	J	U	12.5	9	4.71	5.89	0.757	0.183
116*	PA4352		Universal stress protein	T	C	30.8	34	5.92	5.00	0.618	0.096
**117**	PA4366	*sodB*	Superoxide dismutase	P	P	21.4	21	5.27	5.16	0.835	−0.288
**118**	PA4366	*sodB*	Superoxide dismutase	P	P	21.4	21	5.27	5.00	0.835	−0.288
119	PA4385	*groEL*	GroEL chaperonin	O	C	57.1	58	5.04	5.00	0.831	0.037
120	PA4386	*gorES*	GroES chaperonin	O	C	10.3	11	5.20	5.16	0.692	−0.072
121*	PA4406	*lpxC*	UDP acyl acetylglucosamine deacetylase	M	C	33.4	81	5.21	5.19	0.638	−0.066
**122**	PA4407	*ftsZ*	Cell division protein	D	C	41.2	44	4.93	4.79	0.743	0.016
**123**	PA4407	*ftsZ*	Cell division protein	D	C	41.2	43	4.93	4.86	0.743	0.016
**124**	PA4407	*ftsZ*	Cell division protein	D	C	41.2	43	4.93	4.90	0.743	0.016
125	PA4408	*ftsA*	Cell division protein	D	C	44.6	45	5.20	5.18	0.635	0.038
126	PA4425		Phosphoheptose isomerase	G	C	21.4	24	5.00	4.89	0.658	−0.023
127	PA4431		Iron-sulfur protein	C	CM	20.8	25	6.07	6.01	0.545	−0.092
**128**	PA4450	*murA*	UDP-N-acetylglucosamine carboxyvinyltransferase	M	C	44.6	45	5.52	5.50	0.742	0.152
**129**	PA4450	*murA*	UDP-N-acetylglucosamine carboxyvinyltransferase	M	C	44.6	45	5.52	5.38	0.742	0.152
130	PA4458		Conserved hypothetical protein	R	C	19.3	18	5.52	5.48	0.601	0.012
**131**	PA4483	*gatA*	Glu tRNA amidotransferase	J	U	51.9	52	5.52	5.45	0.638	−0.119
**132**	PA4483	*gatA*	Glu tRNA amidotransferase	J	U	51.9	51	5.52	5.32	0.638	−0.119
**133**	PA4495		Hypothetical protein	S	U	24.9	28	5.79	5.33	0.677	−0.146
**134**	PA4495		Hypothetical protein	S	U	24.9	28	5.79	5.39	0.677	−0.146
135	PA4572	*fklB*	Peptidylprolyl cis-trans isomerase	O	OM	21.8	24	4.78	4.55	0.747	−0.109
136	PA4602	*glyA3*	Serine hydroxymethyltransferase	E	C	45.2	46	5.70	5.71	0.828	−0.087
137	PA4687	*hitA*	Ferric iron-binding protein	P	P	36.1	37	5.54	5.09	0.650	−0.223
138	PA4723	*dksA*	Suppressor protein	T	C	17.3	18	5.04	5.05	0.729	−0.959
139	PA4740	*pnp*	Polyribonucleotide nucleotidyltransferase	J	C	75.5	75	5.07	5.04	0.818	−0.073
140	PA4755	*greA*	Transcription elongation factor	K	C	17.2	20	4.94	4.93	0.769	−0.243
141*	PA4761	*dnaK*	HSP	O	U	68.4	75	4.79	4.75	0.810	−0.326
142*	PA4762	*grpE*	HSP	O	C	20.7	26	4.49	4.36	0.702	−0.604
143*	PA4847	*accB*	Biotin carboxyl carrier	I	U	16.5	22	4.97	4.88	0.741	0.068
144*	PA4886		Two-component sensor	T	CM	50.9	48	6.83	4.92	0.638	−0.004
145	PA4907		Short-chain dehydrogenase	R	C	27.4	28	5.26	5.25	0.696	−0.047
146	PA4920	*nadE*	NH3-dependent NAD synthetase	H	C	29.7	34	5.42	5.59	0.693	−0.193
147	PA4931	*dnaB*	Replicative DNA helicase	L	U	51.6	56	4.98	4.90	0.64	−0.294
148	PA4932	*rplI*	50S ribosomal protein L9	J	C	15.5	13	5.47	5.38	0.726	0.027
**149**	PA4935	*rpsF*	30S ribosomal protein S6	J	C	16.2	14	4.87	4.82	0.830	−1.088
**150**	PA4935	*rpsF*	30S ribosomal protein S6	J	C	16.2	14	4.87	4.73	0.830	−1.088
151	PA4974		Outer membrane protein	MU	OM	53.4	52	5.80	5.40	0.721	−0.518
152*	PA5016	*aceF*	Dihydrolipoamide acetyltransferase	C	C	56.7	67	5.23	5.11	0.694	−0.046
153	PA5040	*pilQ*	Fimbrial biogenesis	U	OM	77.4	74	5.48	5.25	0.621	−0.257
**154**	PA5046		Malic enzyme	C	CM	45.4	44	5.05	4.87	0.736	0.001
**155**	PA5046		Malic enzyme	C	CM	45.4	47	5.05	4.99	0.736	0.001
156*	PA5076		Binding component ABC transporter	ET	P	29.7	27	6.85	6.00	0.770	−0.141
157	PA5110	*fbp*	Fructose bisphosphatase	G	U	37.2	38	5.71	5.75	0.742	−0.274
158*	PA5131	*pgm*	Phosphoglycerate mutase	G	C	55.6	63	5.07	5.06	0.715	−0.118
159	PA5134		Carboxyl-terminal protease	M	U	46.0	48	5.52	5.26	0.734	−0.193
160	PA5140	*hisF1*	Imidazoleglycerol-phosphate synthase	E	C	27.1	28	5.10	5.03	0.628	0.067
161	PA5153		Periplasmic binding protein	ET	P	27.6	27	5.13	4.77	0.795	−0.232
162	PA5161	*rmlB*	dTDP-D-glucose dehydratase	M	U	39.5	40	5.63	5.60	0.640	−0.406
163	PA5171	*arcA*	Arginine deiminase	E	C	46.4	51	5.52	5.47	0.727	−0.219
164	PA5178		Conserved hypothetical protein	S	OM	15.5	12	5.45	5.34	0.698	−0.142
**165**	PA5192	*pckA*	Phosphoenolpyruvate carboxykinase	C	U	55.7	60	5.27	5.23	0.772	−0.152
**166**	PA5192	*pckA*	Phosphoenolpyruvate carboxykinase	C	U	55.7	58	5.27	5.25	0.772	−0.152
167	PA5193	*yrfI*	HSP	O	C	32.8	37	4.73	4.67	0.591	−0.228
168	PA5215	*gcvT1*	Glycine cleavage system protein	E	C	38.9	40	5.43	5.44	0.636	−0.081
169	PA5217		Iron-binding ABC transporter	P	P	36.3	35	6.02	5.73	0.662	−0.233
170	PA5240	*trxA*	Thioredoxin	O	C	11.9	11	4.70	4.62	0.596	−0.117
171	PA5288	*glnK*	Nitrogen regulatory protein	E	C	12.3	9	5.41	5.31	0.676	−0.054
172	PA5312		Aldehyde dehydrogenase	C	C	53.1	55	5.40	5.40	0.762	−0.025
173	PA5321	*dut*	Deoxyuridine triphosphate nucleotidohydrolase	F	U	15.9	12	5.35	5.31	0.687	0.175
174	PA5339		Conserved hypothetical protein	J	U	13.6	10	5.10	5.11	0.759	0.136
175	PA5349		Rubredoxin reductase	C	C	40.6	43	5.50	5.45	0.579	0.087
176	PA5373	*betB*	Betaine aldehyde dehydrogenase	C	C	53.3	56	5.25	5.19	0.636	−0.146
177	PA5429	*aspA*	Aspartate ammonia-lyase	E	C	51.1	49	5.55	5.56	0.758	0.054
178	PA5553	*atpC*	ATP synthase epsilon	C	C	14.7	10	5.14	5.13	0.739	0.158
179	PA5554	*atpD*	ATP synthase beta	C	C	49.5	51	4.98	4.89	0.724	−0.061
180	PA5556	*atpA*	ATP synthase alpha	C	U	55.4	53	5.33	5.24	0.737	−0.073
181	PA5557	*atpH*	ATP synthase delta	C	C	19.3	20	5.78	5.76	0.472	−0.142

† Number on the gel (Fig. S1).

‡ PA number: PAxxxx.

¶ COG functional categories; J, translation, ribosomal structure, and biogenesis; K, transcription; L, DNA replication, recombination, and repair; D, cell division and chromosome partitioning; M, cell envelope biogenesis, outer membrane; N, cell motility and secretion; O, posttranslational modification, protein turnover, chaperones; P, inorganic ion transport and metabolism; T, signal transduction mechanisms; C, energy production and conversion; E, amino acid transport and metabolism; F, nucleotide transport and metabolism; H, coenzyme transport and metabolism; G, carbohydrate transport and metabolism; I, lipid metabolism; Q, secondary metabolites; R, general function prediction only; S, function unknown.

§ Subcellular localization; U, unknown; C, cytoplasm; CM, cytoplasmic membrane; P, periplasm; OM, outer membrane.

†† experimentally derived values.

**xx:** Bold spot number corresponds to protein found in multiple spots.

* : Protein with deviating predicted and experimental p*I* or *M_r_* (underlined).

### Comparison between theoretical and experimental *M_r_* and p*I*

**Figure 3:**
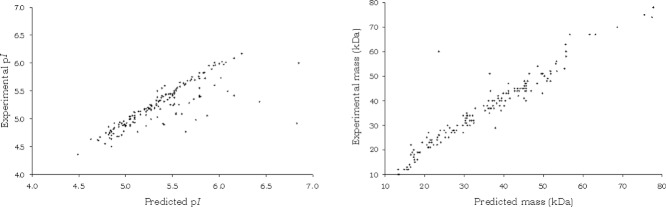
Predicted versus emperimental p*I*- and *M_r_*-values. The scatter plots indicate that predicted versus experimental p*I* (left) and predicted versus experimental mass (right) of identified proteins have a high correlation.

Predicted and experimental p*I* and mass of identified proteins is shown in Table 1. The high correlation between both values for p*I* and *M_r_* is displayed in the scatter plots (Fig. 3).

**Figure 4:**
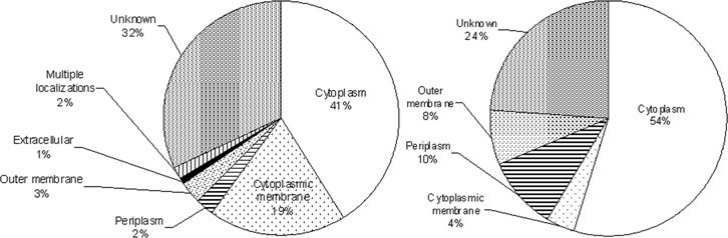
Predicted subcellular localization of the *Pseudomonas aeruginosa* proteome (left) and of the identified proteins (right). Proteins originating from various cellular localizations were identified.

#### p*I*-values

Ninety-three percent of all identified proteins have an experimental p*I* approximating the predicted value. Thirteen proteins have an experimental p*I* that is at least 0.50 units lower than the predicted p*I* (spot numbers marked with an * in Table 1, p*I*-values underlined). The most common modification influencing the proteins’ isoelectric point in prokaryotes is single or multiple phosphorylation (Deutscher and Saier 2005), lowering the p*I* due to the negative charge of the phosphate group. Two-component sensor kinases, such as PA4886 that shows a strong p*I*-shift (–1.91), are known to autophosphorylate (Rodrigue et al. 2000). For some of the proteins with a lowered p*I*-value (PA1084, PA2800, PA5076, and PA0291), a signal peptide was predicted by SignalP. After excluding these amino acids in the sequences, the proteins’ theoretical masses and charges are close to the experimental values, suggesting indeed signal peptide cleavage. The exact nature of the modification can be deciphered by dedicated mass spectrometric analysis, which was beyond the aim of this study.

#### *M_r_*-values

Ninety-seven percent of the identified proteins have an experimental *M_r_* matching the predicted value. Modifications influencing protein mass are isoform splicing or addition of heavy groups, for example, ADP-ribosylation. The coverage of identified peptide fragments was well spread over the complete protein sequence. Four proteins are at least 5 kDa smaller than predicted, probably caused by the removal of a signal peptide, while 13 are larger than predicted, presumably carrying unknown modifications (spot numbers marked with an * in Table 1, *M_r_*-values underlined).

### Protein isoforms

As many as 16 proteins, especially high-abundant proteins, appear as multiple spots on the gel (spot numbers bold in Table 1). Half of these proteins show only a p*I* shift, the other half show both a shift in charge and mass. These spots may be artifacts caused by the high abundance or may be the result of actual posttranslational modification. Little is known, however, about the full extent of protein modification and isoforms in bacteria. SucD, for example, was found in four separate spots (41–44) (Fig. 2), with a p*I* range of 5.42–5.72, while the predicted p*I* is 5.79 (Table 1). Crystal structures have revealed a phosphorylation of SucD in *E. coli* (Wolodko et al. 1994), possibly explaining the lowered p*I*-value of the highly similar SucD in *P. aeruginosa*.

### Subcellular localization and GRAVY

All annotated *P. aeruginosa* PAO1 proteins were classified according to their predicted localization (PseudoCAP) (Fig. 4). This calculation shows that 41% of the proteome is localized in the cytoplasm, 19% is directed to the cytoplasmic membrane. A small fraction is transported to the periplasm (2%), the outer membrane (3%), or the extracellular environment (1%). The remaining one-third of the proteins cannot be localized based on their amino acid composition. This distribution of proteins at each localization is consistent across species, independent of proteome size (Gardy et al. 2005).

Among the 159 identified proteins, no extracellular proteins are found. This is not surprising since these are most likely discarded along with the growth medium during sample preparation. Outer membrane proteins and periplasmic proteins are present (12 and 16, respectively), but cytoplasmic membrane proteins are considerably underrepresented (6), consistent with the assumption that integral membrane proteins have low solubility near their isoelectric point and are thus difficult to detect under standard 2-DE conditions. The GRAVY value predicts the hydrophobicity of a protein: hydrophobic membrane proteins are believed to have a positive value. Therefore, GRAVY values ought be linked to the subcellular localization. The calculation of the mean GRAVY values confirms this assumption for *P. aeruginosa*. The mean value of the total *P. aeruginosa* proteome is –0.075. Predicted inner membrane proteins have a significantly (*P* < 0.0001) higher GRAVY value (0.448) than predicted intracellular proteins (–0.193). Periplasmic and outer membrane proteins, on the other hand, typically have negative GRAVY values.

The identified proteins have a mean GRAVY value of –0.129, which is slightly lower than the total proteome value (*P* < 0.05). Among these proteins, only one has a GRAVY value above 0.400 (PA4053, spot 106). Therefore, the underrepresentation of cytoplasmic membrane proteins is assumed to be caused by their high hydrophobic nature and by the chosen p*I* range.

### Abundance and CAI

The CAI is a measure of how well a gene is adapted to the translational machinery. In general, a high CAI (>0.70) suggests a high expression level (Gasteiger et al. 2005). Using strongly expressed genes as codon usage template, the mean CAI of all *P. aeruginsoa* PAO1 genes is 0.58 (Grocock and Sharp 2002), the mean value for the identified proteins is 0.70. However, when plotting spot volumes against protein CAI-values, the correlation is surprisingly low (*R*^2^ = 0.021). Nevertheless, this is in agreement with the observation of Grocock and Sharp (2002), who pointed out that the CAI appears to be a poor statistic for organisms with a biased base composition, such as *P. aeruginosa* that has a GC-content of 67%.

### Functional classification

All bacterial proteomes present in the public databases, including *P. aeruginosa*, were classified in COG protein categories, representing major biological cell functions (Tatusov et al. 1997). The protein distribution seems to be fairly similar for all bacteria, and no COG category appears to be overrepresented in the large *P. aeruginosa* proteome (http://www.ncbi.nlm.nih.gov/sutils/coxik.cgi?gi=163).

The 159 identified proteins represent every existing COG category (Table 1). Even some low-abundant signaling proteins were identified, indicating a good representation of the total proteome on the 2-DE gel. Half of the identified proteins are important for metabolism, particularly energy conversion and amino acid metabolism. One-quarter functions in cellular processes, for example, protein turnover or cell envelope biogenesis. Other proteins play a role in translation or are poorly characterized.

The majority of identified proteins, which included large spots, function in carbohydrate metabolism and energy production. These represent enzymes from major biochemical pathways such as oxidative phosphorylation (7), reductive carboxylate pathway (4), pentose phosphate pathway (4), carbon fixation (6), citrate cycle (6), glycolysis and gluconeogenesis (6). This high representation suggests a strong expression of these key enzymes. Other major identified proteins on the 2-DE gel correspond to chaperones (GrpE, GroEL, GroES, trigger factor, and DnaK) responsible for proper folding of newly formed proteins. Protein chaperones and energy-conversion enzymes also appear as intense spots on other bacterial 2-DE maps (Wolodko et al. 1994; Rodrigue et al. 2000; Gardy et al. 2005).

### Hypothetical proteins

Apart from the classified proteins, 19 spots correspond to proteins marked as hypothetical in the *Pseudomonas* database, 12 of which so far lacked experimental confirmation (PA0446, PA0664, PA0976, PA1597, PA1677, PA1837, PA2806, PA3302, PA3481, PA3801, PA4458, and PA5339). Among those 19 proteins, 12 are conserved in other organisms. Obviously, their substantial expression suggests that they have biological roles in *P. aeruginosa*, which are thus far elusive. Their presence on a 2-DE gel opens perspectives for comparative studies.

## Conclusions

We report a proteome analysis of *P. aeruginosa* PAO1, a species representing many strains of either clinical or environmental importance. The theoretical and experimental proteomes were compared by generating a 2-D reference map. On this map focused on cytoplasmic proteins, 181 spots were identified as corresponding to 159 different protein entries. Despite the low amount of hydrophobic proteins, these results show that the spots on the 2-DE map form a satisfactory and representative subset of the *P. aeruginosa* proteome; proteins from all predicted subcellular localizations and all functional categories are detected and identified. Moreover, 19 proteins, so far classified as hypothetical, are now experimentally confirmed. The data provide a reference for subsequent comparative studies of the biology and metabolism of *P. aeruginosa*, aimed at unraveling global regulatory networks.

## Supporting information

**Figure S1**. Two-dimensional gel electrophoresis (2-DE) reference map of the *Pseudomonas aeruginosa* proteome.
