# Supplementary figures and images for: A theoretical and experimental proteome map of Pseudomonas aeruginosa PAO1
Source: Microbiologyopen. 2012 Jun;1(2):169–81. doi: 10.1002/mbo3.21 (PMC3426416; doi:10.1002/mbo3.21)

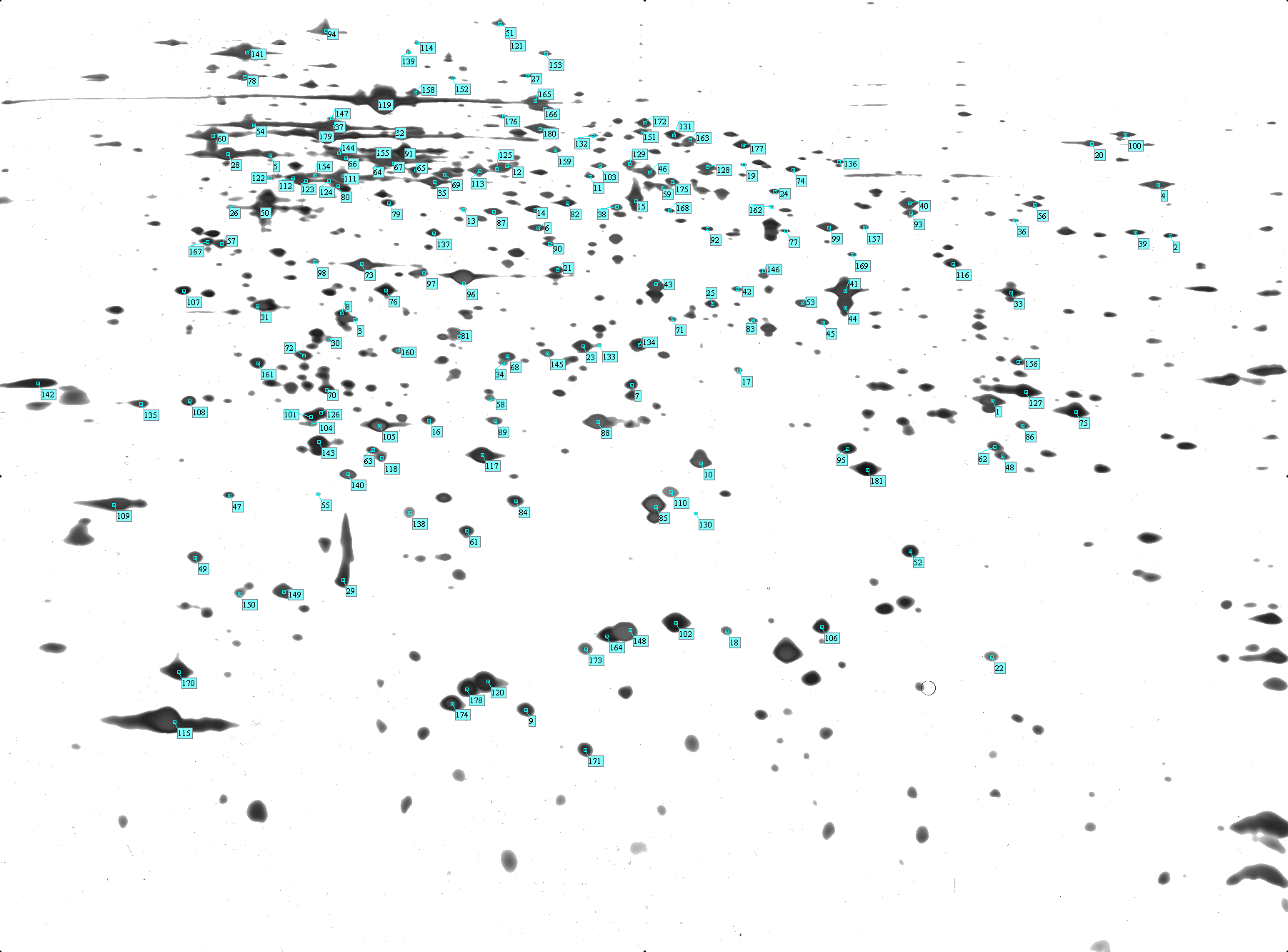

Supplement: Supplementary file 1 [file mbo30001-0169-SD1.tif]
